# Persistent wandering atrial pacemaker after epinephrine overdosing – a case report

**DOI:** 10.1186/1471-2431-13-1

**Published:** 2013-01-02

**Authors:** Elhadi H Aburawi, Hassib Narchi, Abdul-Kader Souid

**Affiliations:** 1 Department of Pediatrics, College of Medicine and Health Science, United Arab Emirates University, P. O. Box 17666, Al-Ain, UAE

**Keywords:** Epinephrine, Iatrogenic, Supraventricular tachycardia, Sympathomimetic toxicity, Wandering pacemaker, Arrhythmia

## Abstract

**Background:**

Long-term complications of sympathomimetic drug overdosing have not been adequately investigated in infants and young children. Despite reports discouraging their use in children, these formulations are frequently administered for “cold-like symptoms”. Their frequent adverse events are different forms of arrhythmias, including multifocal atrial tachycardia.

**Case presentation:**

A 3-year-old toddler developed multifocal atrial tachycardia following an iatrogenic overdose of epinephrine accidentally administered intravenously. His ECG showed wandering atrial pacemaker (p-waves with different origins and configurations) that persisted for at least one year. This event demonstrated the sensitivity of young children to the sympathomimetic drugs, especially overdosing.

**Conclusions:**

Health care providers and parents should be warned of toxicities associated with sympathomimetic drug overdosing. Future studies are needed to determine whether wandering atrial pacemaker is a potential long-term complication of high-dose sympathomimetics.

## Background

Sympathomimetic drugs are known to induce arrhythmias, including multifocal atrial tachycardia, re-entry tachycardia, atrial fibrillation and wandering atrial pacemaker [1,2]. The mechanism is thought to be due to sinus node and atrial tissue sensitivity to α- and β-adrenergic stimuli as well as to decreased cardio-vagal reflex. The mechanisms involve autonomic transmitters, changes in impulse formation, conduction and refractoriness [3,4]. Supraventricular tachycardia may induce left ventricular dysfunction and cardiomyopathy, which are usually reversible once the tachycardia resolves [5].

Multifocal atrial tachycardia and wandering pacemaker have already been reported in association with sympathomimetic drug overdosing [2,6]. Multifocal (chaotic) atrial tachycardia is defined as multiple distinct P-wave morphologies, irregular P-P intervals and isoelectric baseline between P-waves with a rapid ventricular rate. Patients with a wandering atrial pacemaker are usually asymptomatic and have irregularly irregular rhythm. The normal heart rate in wandering atrial pacemaker differentiates this condition from multifocal atrial tachycardia [2,6]. Multifocal atrial tachycardia has been described in association with respiratory viruses [7]. The fate of the aberrant atrial electrical activity is unknown.

This report describes a toddler with iatrogenic intravenous epinephrine overdose. This accidental exposure resulted in a lasting wandering atrial pacemaker.

## Case presentation

A previously healthy 3-year-old boy (weight 17.5 kg and height 102.5 cm) presented with viral laryngo-tracheobronchitis (croup). His initial heart rate was 120 beats per minute (bpm). One milligram epinephrine was administered via a nebulizer without significant changes in his heart rate. As respiratory symptoms persisted, a second dose of epinephrine (1 mg) was prescribed, but was inadvertently administered by intravenous push into a peripheral line. This amount was approximately 6-times the recommended dose (0.01 mg/kg body weight). The child suddenly developed facial flushing, tachycardia (187 bpm), hypertension (110/75 mmHg), hypoxemia (O_2_ saturation 90% in room air) and worsening of his dyspnea. There was neither gallop nor hepatomegaly. Chest x-ray showed increased vascular markings, which improved after a dose of furosemide.

**Figure 1:**
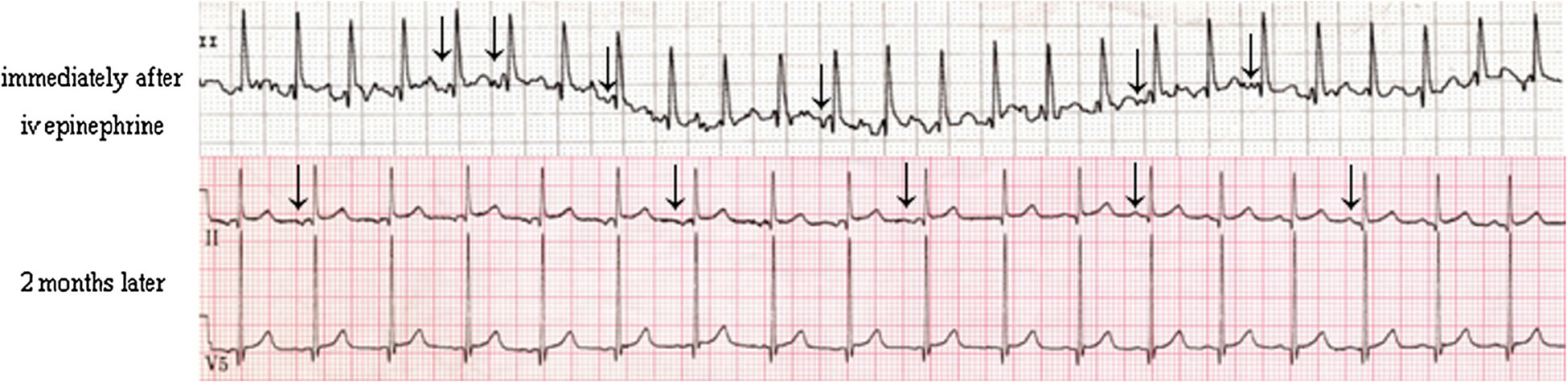
**ECG findings.***Upper panel*, rhythm strip (lead II) immediately after the intravenous injection of epinephrine, showing a heart rate of 187 bpm, multifocal atrial tachycardia and ST elevation of 4 mm. Notice the different P-wave morphologies; flat (junctional), negative and positive p-waves are evident (arrows). *Lower panel*, ECG (leads II and V5) two months later showing a ventricular rate of 115 bpm and persistent wandering atrial pacemaker. The same findings were present at 12 and 18 months.

The child developed multifocal atrial tachycardia with multiple distinct P-wave morphologies, irregular P-P intervals, isoelectric baseline between P-waves and rapid ventricular rate (187 bpm). The QRS complex ≤ 100 ms and ST-segment elevation ≥ 3 mm were evident on the rhythm strip (Figure 1, upper panel). Tachycardia persisted for about 12 hours although the wandering pacemaker persisted after normalization of the heart rate. This was confirmed on 24-hour Holter tape recording from 12 to 36 hour after the IV epinephrine administration; the ventricular rate and QRS duration were normal. ECG (Figure 1, lower panel) and 24-hour Holter tape recording at 2, 12 and 18 months showed persistent wandering pacemaker with normal QRS duration and ventricular rate (about 100 bpm).

During the acute episode, echocardiogram showed normal heart structure and ventricular function. Serum Troponin-T and creatine kinase-MB were also normal. His vital signs (including heart rate and blood pressure), O_2_ saturation and ECG were closely monitored. His blood pressure normalized 4 hours after the epinephrine dose. He received a dose of intravenous furosemide and oxygen supplementation. He was discharged after 48 hours with normal vital signs.

Another cardiac event noted in this patient was an elevated ST-segment (~4 mm, a sign of coronary spasm), Figure 1.

Fleming et al. have published evidence-based centile charts from birth to 18 years of age for normal heart rates [8]. Between 1 and 5 years, the heart rate (mean ± SD) is 109 ± 14 bpm for males and 108 ± 15 bpm for females. The corresponding values for infants are 132 ±12 bpm and 135 ± 14 bpm [9]. Thus, the heart rate noted for this patient (187 bpm) was 5 SD above the mean.

Children with marked tachycardia (≥4 SD) should be monitored (vital signs, pulse oximetry and ECG). Serum troponin-T, serum creatine kinase-MB and chest x-ray may be necessary based on clinical and ECG findings. Echocardiography is also needed to rule out underlying cardiac pathology. Treatment with adenosine, beta-blockers or calcium-channels blockers should be individualised. Therapeutic interventions are justified in symptomatic children (e.g., heart failure, chest pain and dyspnea), persistent tachy-arrhythmia, ischemic changes on ECG or abnormal serum cardiac biomarkers. In specific cases, electrophysiological studies may be necessary to rule out sinus node re-entry or right atrial tachycardia. Follow-up by a pediatric cardiologist may be necessary, especially in the presence of abnormal ECG.

## Conclusions

This report illustrates the potential seriousness of sympathomimetic overdosing. Young children are especially vulnerable due to high-sensitivity of their sinoatrial node and atrial tissue to catecholamines. The wandering atrial pacemaker in this patient could be a long-term consequence, since it persisted at least 18 months after the incident.

The epinephrine overdose as the cause of his p-wave abnormality (wandering atrial pacemaker) could not be confirmed: as he had no ECG prior to the episode, this finding may have existed before the administration of epinephrine. We can only speculate that the multifocal atrial tachycardia present during the acute episode, instead of resolving, persisted and lead to a wandering atrial pacemaker. This sequel could result from a significant damage to the SA-node or atrial tissue, which persisted for at least 18 months.

Sympathomimetics overdosing might be relatively common in infants and children, since these medications are frequently used in emergency settings and also at home because of their availability “over-the-counter”. Health care providers and parents should be warned of toxicities associated with sympathomimetic drugs. Future studies are needed to determine whether wandering atrial pacemaker is a potential long-term complication of high-dose sympathomimetics.

## Competing interests

There are no potential, perceived, or real conflicts of interest. No financial or non-financial interests.

## Authors’ contributions

All authors have read and approved the final manuscript. EHA collected and analyzed the patient’s data, including the ECG and played a major role in writing the manuscript; HN aided in the editing of the manuscript. A-KS played a major role in writing the manuscript.

## Pre-publication history

The pre-publication history for this paper can be accessed here:

http://www.biomedcentral.com/1471-2431/13/1/prepub

## References

[B1] Daubert GP Mabasa VH Leung VW Aaron C Acute clenbuterol overdose resulting in supraventricular tachycardia and atrial fibrillation J Med Toxicol 2007 3 56 60 10.1007/BF03160909 18072161 PMC3550084

[B2] Altee JL 3rd Malkinson BA Potentiation by thiopental of halothane-epinephrine-induced arrhythmias in dogs Anesthesiology 1982 57 285 288 10.1097/00000542-198210000-00006 7125265

[B3] Antoni H Pathophysiology of cardiac arrhythmias involving autonomic transmitters Z Kardiol 1986 75 Suppl 5 1 8 2881402

[B4] Schlepper M Effects of the autonomic nervous system in supraventricular arrhythmia Z Kardiol 1986 75 Suppl 5 35 40 3030014

[B5] Packer DL Bardy GH Worley SJ Smith MS Cobb FR Coleman RE Gallagher JJ German LD Tachycardia induced cardiomyopathy: a reversible form of left ventricular dysfunction Am J Cardiol 1986 57 563 570 10.1016/0002-9149(86)90836-2 3953440

[B6] Maupoil V Ectopic activity in the rat pulmonary vein can arise from simultaneous activation of α1- and β1-adrenoceptors Br J Pharmacol 2007 150 899 905 10.1038/sj.bjp.0707177 17325650 PMC2013875

[B7] Wu MY Wu ZF Chen XY Chaotic atrial tachycardia in 22 infants Chin Med J 1984 97 500 503 6441685

[B8] Fleming S Thompson M Stevens R Heneghan C Plüddemann A Maconochie I Tarassenko L Mant D Normal ranges of heart rate and respiratory rate in children from birth to 18 years of age: a systematic review of observational studies Lancet 2011 377 1011 1018 10.1016/S0140-6736(10)62226-X 21411136 PMC3789232

[B9] Salameh A Gebauer RA Grollmuss O Vít P Reich O Janousek J Normal limits for heart rate as established using 24-hour ambulatory electrocardiography in children and adolescents Cardiol Young 2008 18 467 472 18634710 10.1017/S1047951108002539

